# Automated Gait Analysis Based on a Marker-Free Pose Estimation Model

**DOI:** 10.3390/s23146489

**Published:** 2023-07-18

**Authors:** Chang Soon Tony Hii, Kok Beng Gan, Nasharuddin Zainal, Norlinah Mohamed Ibrahim, Shahrul Azmin, Siti Hajar Mat Desa, Bart van de Warrenburg, Huay Woon You

**Affiliations:** 1 Department of Electrical, Electronic and Systems Engineering, Faculty of Engineering and Built Environment, Universiti Kebangsaan Malaysia, Bangi 43600, Malaysia; tonyhiiwork@gmail.com (C.S.T.H.); nasharuddin.zainal@ukm.edu.my (N.Z.); 2 Neurology Unit, Department of Medicine, Faculty of Medicine, Universiti Kebangsaan Malaysia Medical Centre, Kuala Lumpur 56000, Malaysia; norlinah@ppukm.ukm.edu.my (N.M.I.); shahrulazmin@gmail.com (S.A.); thesitihajar1@gmail.com (S.H.M.D.); 3 Department of Nursing, Hospital Canselor Tuanku Muhriz, Kuala Lumpur 56000, Malaysia; 4 Department of Neurology, Radboud University Medical Center, P.O. Box 9101, 6500 HB Nijmegen, The Netherlands; bart.vandewarrenburg@radboudumc.nl; 5 Pusat GENIUS@Pintar Negara, Universiti Kebangsaan Malaysia, Bangi 43600, Malaysia; hwyou@ukm.edu.my

**Keywords:** automated gait analysis, pose estimation, temporal gait parameters, markerless

## Abstract

Gait analysis is an essential tool for detecting biomechanical irregularities, designing personalized rehabilitation plans, and enhancing athletic performance. Currently, gait assessment depends on either visual observation, which lacks consistency between raters and requires clinical expertise, or instrumented evaluation, which is costly, invasive, time-consuming, and requires specialized equipment and trained personnel. Markerless gait analysis using 2D pose estimation techniques has emerged as a potential solution, but it still requires significant computational resources and human involvement, making it challenging to use. This research proposes an automated method for temporal gait analysis that employs the MediaPipe Pose, a low-computational-resource pose estimation model. The study validated this approach against the Vicon motion capture system to evaluate its reliability. The findings reveal that this approach demonstrates good (ICC_(2,1)_ > 0.75) to excellent (ICC_(2,1)_ > 0.90) agreement in all temporal gait parameters except for double support time (right leg switched to left leg) and swing time (right), which only exhibit a moderate (ICC_(2,1)_ > 0.50) agreement. Additionally, this approach produces temporal gait parameters with low mean absolute error. It will be useful in monitoring changes in gait and evaluating the effectiveness of interventions such as rehabilitation or training programs in the community.

## 1. Introduction

A person’s gait is referred to as his or her style or pattern of walking. Numerous factors, such as sex [1], age [2,3], walking speed [1,4,5], and type of disease, can affect gait. As gait patterns can be quite characteristic in certain disorders, such as the typical shuffling gait in parkinsonism, analyzing gait patterns can be very helpful in establishing medical diagnoses. Gait analysis has numerous applications in different fields, including clinical biomechanics, rehabilitation [6,7,8], sports science [9], robotics [9], ergonomics, and forensics [1,10]. Gait analysis is a critical tool in understanding the complexities of human movement and diagnosing movement-related conditions. It enables clinicians and researchers to identify biomechanical abnormalities, evaluate the effectiveness of treatment plans, develop personalized rehabilitation programs, and improve athletic performance. By understanding the nuances of an individual’s gait, it is possible to improve mobility, reduce the risk of injury, and enhance quality of life.

In the past, gait evaluation relied heavily on visual analysis [11], but more recently, instrumented gait analysis has gained popularity. This shift is driven by the shortcomings of visual gait analysis, including its low inter-rater reliability and dependence on clinical expertise. Instrumented gait analysis involves using specialized equipment, such as motion capture systems [12,13], force plates [14], electromyography [15], pressure sensors [16], and inertial measurement units (IMU) [7], to measure and analyze the gait parameters quantitatively. These systems can provide objective and quantifiable data on various parameters of gait, such as joint angles, range of motion, and gait speed. However, instrumented gait analysis is expensive, invasive, time-consuming, and requires specialized equipment and trained personnel. As a result, it is typically used in research laboratories or clinical settings, and it may not be accessible to the general public. Therefore, further research into markerless gait assessment systems is necessary.

With the advancement of computer vision techniques, it is now possible to conduct markerless gait analysis based on video footage by utilizing pose estimation models such as OpenPose [17], BlazePose [18], and YOLO-Pose [19]. Pose estimation techniques in markerless gait analysis involve using computer vision and machine learning algorithms to extract human poses from video footage and track the movement of the body’s joints and limbs in 2D or 3D spaces. These techniques typically involve identifying key points on the body, such as the joints, and then tracking their movements over time. By analyzing movement patterns, it is possible to determine various gait parameters, such as step length, walking speed, and joint angles [20]. Additionally, 3D camera technologies, such as Microsoft’s Kinect [21,22,23,24] and Intel RealSense cameras [25], use depth sensors and infrared cameras to create a 3D representation of a human skeleton in almost real-time. While these 3D approaches have shown to be effective in some spatiotemporal gait outcomes, their high cost (due to the requirement for specialized hardware rather than a generic camera) compared to 2D-based deep learning approaches restricts their use in gait analysis. In addition, a study revealed that Microsoft Kinect’s ability to track posture is limited as it can accurately assess the knee joint angle only when the knees are visible and not covered by clothing [26]. Another research study found that the accuracy of gait analysis results using the foot velocity method and knee distance method, based on Microsoft Kinect technology, is notably influenced by the subject’s clothing. However, the accuracy of the center height method was not affected by clothing [27].

Up to now, the research on 2D markerless pose estimation has concentrated on assessing normal walking or gait. Several studies have proved that this technique can assess gait kinematics, temporal gait parameters, and spatial gait parameters, and the results have generally been positive [26,28,29,30,31]. Viswakumar et al. [26] suggested a technique for estimating poses in two dimensions without using markers, which relies on the OpenPose pose estimation model and a basic mobile phone camera to compute the knee flexion angle with minimal mean absolute standard deviation. D’Antonio et al. [28] suggested an inexpensive, markerless system for detecting and tracking human motion in 3D, which uses OpenPose, two webcams, and a linear triangulation algorithm to monitor lower limb motion in a manner comparable to IMUs. Stenum et al. [29] proposed a 2D video-based approach for analyzing human gait using an OpenPose pose estimation model to evaluate spatiotemporal and sagittal kinematic gait parameters with low mean absolute error. Viswakumar et al. [30] introduced an OpenPose-based, markerless gait analysis system capable of assessing lower joint kinematics with low mean error. Tony Hii et al. [31] conducted a comparison of three markerless pose estimation models (OpenPose, MMPose, and MediaPipe Pose) in terms of their ability to assess lower limb kinematics, and determined that MediaPipe Pose is the optimal model for evaluating lower limb joint kinematics. However, most gait analysis using markerless pose estimation techniques relies heavily on OpenPose, a pose estimation model that requires significant computational resources, which limits its use in natural environments such as homes or outdoor settings. Additionally, markerless pose estimation-based gait analysis requires human intervention to produce temporal and spatial gait outcomes, which is not very user-friendly.

This study proposes a method for automated temporal gait analysis using the MediaPipe Pose (3D top-down pose estimation model) with a single camera for running. The objective of the research is to evaluate and validate the reliability of this automated temporal gait analysis method compared to a reference-standard 3D Vicon motion capture system. The study involved analyzing a freely available dataset that includes synchronized digital video recordings of walking sequences and three-dimensional motion capture gait data [32]. The digital video recordings of walking sequence were initially analyzed using the MediaPipe Pose model to identify the location of each body joint in each video frame. An algorithm was then applied to automatically detect specific gait events, such as heel-strike and toe-off, based on the joint locations obtained. Temporal gait parameters were calculated using the detected gait events and compared to measurements obtained from the Vicon motion capture system, which is widely regarded as the gold standard for gait analysis.

The main contributions of this paper are outlined as follows:

1. A markerless pose estimation model, MediaPipe Pose, which requires lower computational resources, was applied for the extraction of body key points from healthy individuals with promising accuracy and reduced inference speed.
2. An algorithm was devised to automate the assessment of gait parameters based on the body key points extracted using MediaPipe Pose, eliminating the requirement for human intervention.

## 2. Materials and Methods

### 2.1. Dataset

In this research, a dataset that featured synchronized and calibrated video from multiple angles and motion capture was utilized [32]. The dataset was accessible to the public at http://bytom.pja.edu.pl/projekty/hm-gpjatk/ (accessed on 9 September 2022). It included three-dimensional motion capture data and walking video recordings of 32 healthy individuals, comprising 10 females and 22 males. The dataset did not contain identifiable information about the individuals, and the faces of the individuals in the video recordings were blurred.

This study included adult participants between the ages of 20 and 65 who did not have any injuries or conditions that could impact their ability to participate. However, individuals who relied on walking aids or had cognitive issues that could interfere with the study were excluded from participation. Additionally, pregnant women and children were also excluded from participation.

### 2.2. Video Data Collection

**Figure 1:**
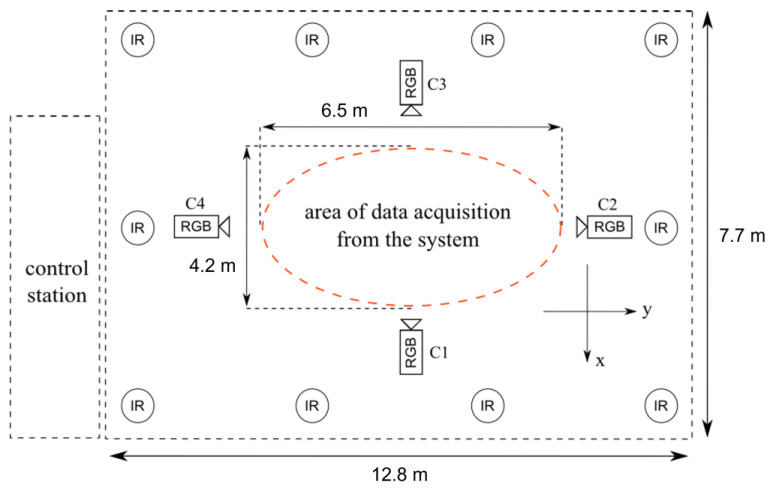
Camera setup for video and Vicon motion capture data collection [32]. (Remarks: RGB: digital video camera, IR: motion capture camera).

For the purpose of recording videos of healthy individuals walking, a setup of four calibrated and synchronized digital video cameras (Basler Pilot piA1900-32gc, Ahrensburg, Germany) was utilized (Figure 1). A subset of the dataset identified as walking sequence s3, which featured a single pathway of 6.5 m, was used for this study. In this subset, individuals were recorded walking diagonally from right to left. The video recordings were captured at a resolution of 960 × 540 pixels and 25 frames per second (fps). However, the data of one healthy individual (data sequence id: p16s3) were excluded as they belonged to another subset (walking sequence s4: walking diagonally from left to right). As a result, a total of 31 healthy individuals were assessed in this research. 

The study opted to use gait analysis based on videos captured from the side view, known as sagittal plane analysis, due to its advantages over frontal plane analysis. One advantage is that sagittal plane analysis is less susceptible to errors caused by changes in camera angle and position compared to frontal plane analysis. Another advantage is that it provides a clear and straightforward representation of the main gait features and overall gait pattern. To ensure accuracy, the study used video recordings captured from the right view using a specific digital camera labeled ‘C1’, which was found to provide more precise gait parameters for all gait cycles compared to recordings from the left view digital camera ‘C3’, as suggested by Stenum et al. [29].

### 2.3. Reference Standard—Vicon 3D Motion Capture System

**Figure 2:**
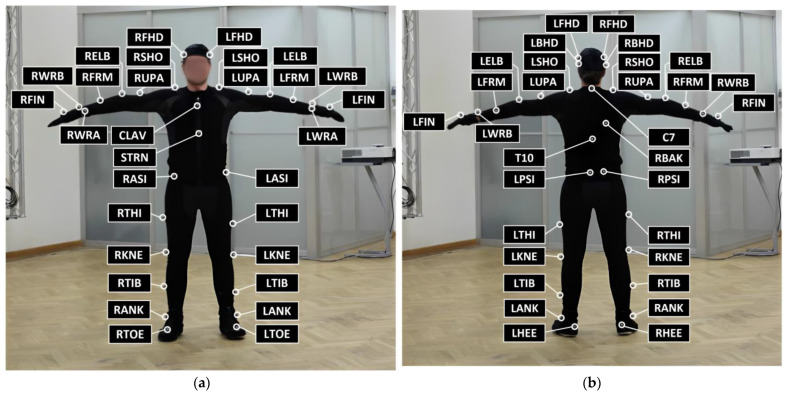
Retroreflective spherical marker placement on the anatomical landmarks of the healthy individual for (**a**) front view, (**b**) back view [32].

In order to evaluate the accuracy and reliability of our system, a 3D motion tracking system was established, utilizing ten motion capture cameras (Vicon MX-T40, Denver, CO, USA) to generate a 3D skeleton of individuals while walking (Figure 1). Prior to the walking test, the healthy individual was instructed to wear 39 retroreflective spherical markers on specific anatomical landmarks (Figure 2). Out of these markers, 4 were placed on the head, 14 on the arms, 12 on the legs, 5 on the torso, and 4 on the pelvis. The Vicon Motion Capture (moCap) system, consisting of ten MX-T40 cameras with a resolution of 2352 × 1728 pixels, tracked the moCap data (i.e., 3D positions of markers) at a frequency of 100 Hz. The gait analysis tool included in the Vicon system was utilized to produce a set of gait results, which were used as a benchmark for comparison purposes.

### 2.4. Pose Estimation Model for Gait Assessment

**Figure 3:**
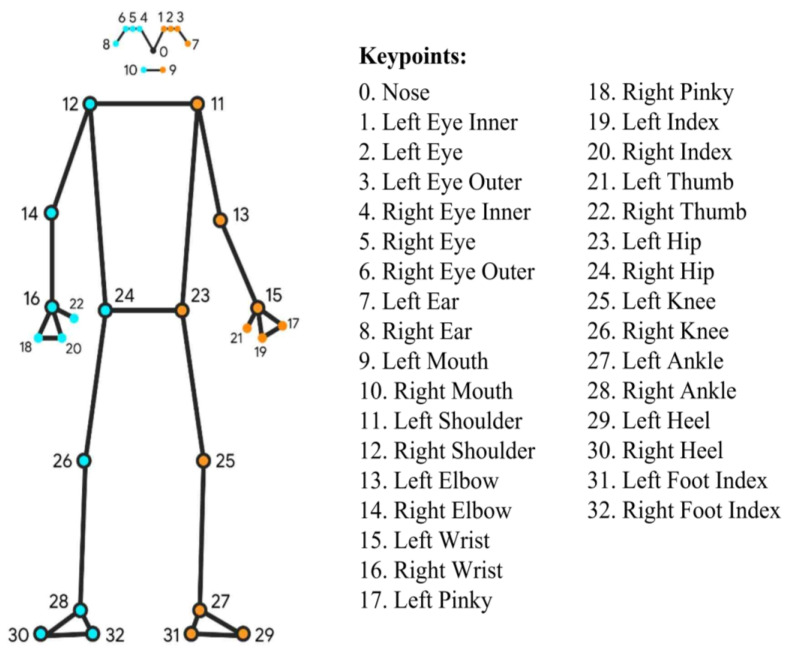
33 anatomical landmarks/body key points extracted from MediaPipe Pose [18].

MediaPipe Pose is a Google-developed machine learning (ML) technology that uses RGB video frames to track the body pose of an individual by identifying 33 three-dimensional anatomical landmarks/body key points (Figure 3). It is built on the BlazePose research [18] that also powers the ML Kit Pose Detection API (a lightweight solution for app developers to detect body poses in real-time). It is known for its low computation cost [18,31], which allows for real-time pose tracking, and its cross-platform compatibility. This makes it suitable for deployment on various devices, such as mobile phones, desktops/laptops, and even on the web, and in programming languages such as Python. There are three models (BlazePose GHUM Heavy, BlazePose GHUM Full, and BlazePose GHUM Lite) available for pose estimation in MediaPipe Pose, and for this study, the BlazePose GHUM Heavy model was chosen for gait assessment in the sagittal plane due to its accurate estimation of body key points. The minimum confidence levels for human tracking and key points detection were set at 0.5.

### 2.5. Gait Parameter Extraction

**Figure 4:**
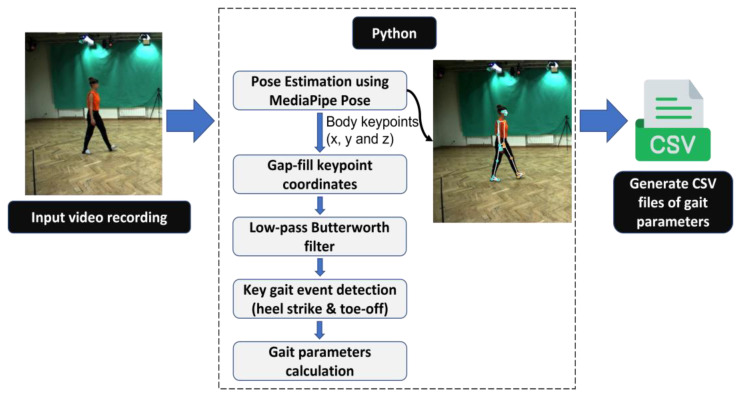
The workflow of the proposed approach based on MediaPipe Pose.

The proposed system utilized the Python application installed on a laptop (12th Gen Intel Core i7-12700H CPU) to conduct the temporal gait analysis based on the 3D markerless pose estimation model (MediaPipe Pose). The system inputted the walking video recorded using a digital video camera labeled ‘C1’ and outputted the temporal gait analysis results in a comma-separated values (CSV) file for each healthy individual (Figure 4). The pseudo-code for the proposed system is presented in Algorithm 1. The following section details how temporal gait parameters are extracted from the X, Y, and Z locations of body key points utilizing signal analysis.
**Algorithm 1** Pseudo-code for the Proposed System**Input:** Walking video of healthy individual **Output:** Gait parameters results in CSV file **Begin**1   Initialize MediaPipe Pose Estimator2   **while** (current video frame ≤ last video frame) **do**3       Identify the region-of-interest that contains human pose4       Extract and save the positions of body keypoints in the region-of-interest5    **end while**6    Gap-filled-body-keypoints = Gap-fill (body keypoints)7    Setup 10th order Butterworth low pass filter at normalized cut off frequency = 0.17528    Filtered-body-keypoints = Butterworth-low-pass-filter (Gap-filled-body-keypoints)9    Calculate the relative changes in distance between the hip and foot-index for the left and right legs over time10   Identify the peak and minima of the relative changes in distance between the hip and foot-index for the left     and right legs over time11   Heel-strike-event-timings-left-leg = Timings of peak occurrence (left leg)12   Heel-strike-event-timings-right-leg = Timings of peak occurrence (right leg)13   Toe-off-event-timings-left-leg = Timings of minima occurrence (left leg)14   Toe-off-event-timings-right-leg = Timings of minima occurrence (right leg)15   Stance-time = Time duration between heel strike and toe-off of the same leg16   Swing-time = Time duration between toe-off and heel-strike of the same leg17   Step-time = Time duration between consecutive heel strikes of both feet18   Double-support time = Time duration between heel-strike of one leg and toe-off of the contralateral leg19   Save Stance-time, Swing-time, Step-time, Double-support-time in CSV file**End**

#### 2.5.1. Pose Estimation Using MediaPipe Pose

The pose estimation process using MediaPipe Pose involved a well-established two-step detector–tracker machine learning pipeline. In the first step, the detector identified the region-of-interest (ROI) containing the pose within each frame. Then, in the second step, the tracker extracted the positions of all 33 pose key points within this ROI. Each pose’s key points included the following information:

1. x and y: The coordinates of the key points, normalized to a range of [0.0, 1.0] based on the image width and height, respectively.
2. z: The depth of the key points relative to the midpoint of the hips, where smaller values indicated proximity to the camera. The scale of z was roughly comparable to x.
3. Visibility: A value ranging from 0.0 to 1.0, indicating the likelihood of the key points being visible and unobstructed in the image.

**Figure 5:**
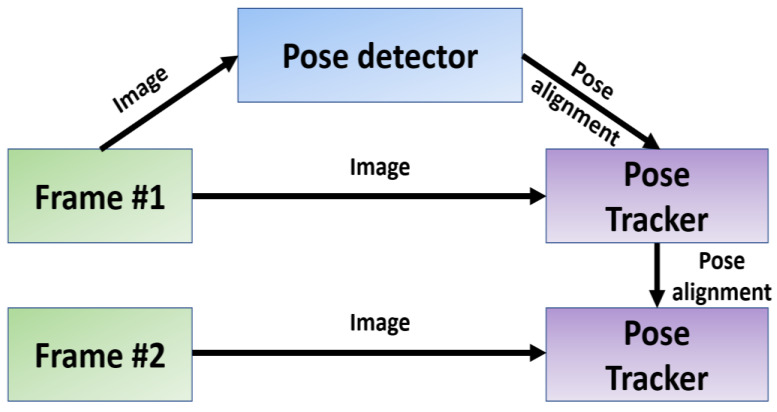
The pipeline overview for human pose estimation using MediaPipe Pose.

It is worth noting that, in the case of video, the detector was only applied to the first frame. For subsequent frames, the ROI was derived from the previous frame’s pose ke points, as depicted in Figure 5.

#### 2.5.2. Data Preprocessing (Gap Filling and Low Pass Filtering)

The body key points location data (x, y, and z coordinates) extracted using MediaPipe Pose was then gap-filled using cubic spline interpolation. The 10th-order Butterworth low pass filtering with a normalized cut-off frequency of 0.1752 was then applied to remove any spikes in the data series. This data preprocessing could reduce the noise that was not indicative of the real position of the body key points location extracted by MediaPipe Pose.

#### 2.5.3. Temporal Gait Parameters Extraction: Identifying Key Gait Events

**Figure 6:**
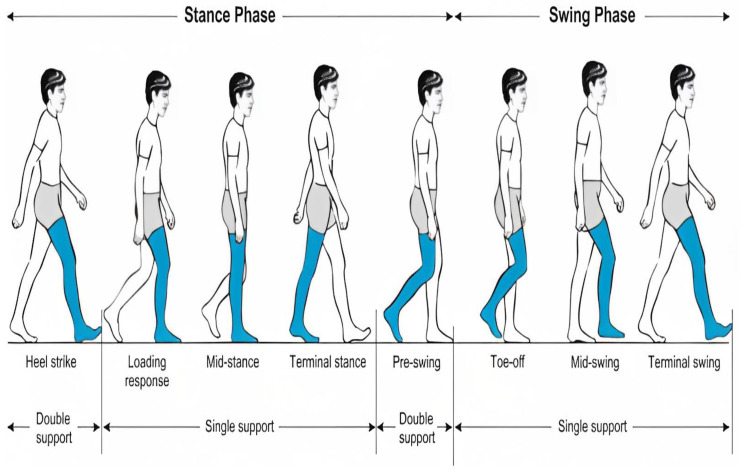
Phases of the normal gait cycle [33].

Heel strike and toe-off are the key gait events that aid the extraction of the temporal gait characteristics. Based on the gait cycle in Figure 6, the heel strike event occurs when the foot index is farthest forward (maximum relative distance between hip and foot index) while the toe-off event occurs when the foot index is farthest backward (minimum relative distance between hip and foot index). 

**Figure 7:**
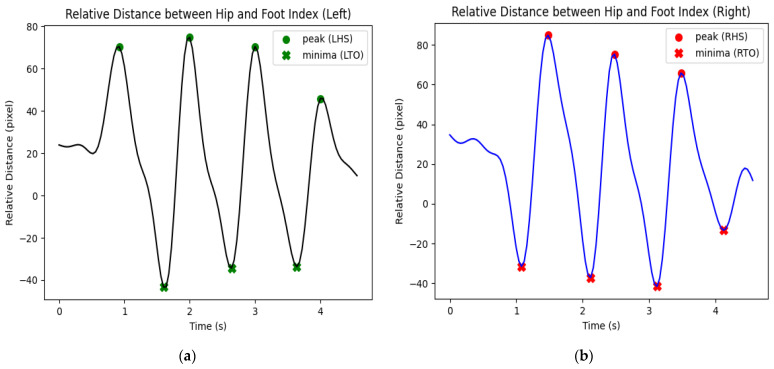
The relative distance between hip and foot index for: (**a**) Left leg; (**b**) Right leg. (Remarks: LHS: left heel strike, LTO: left toe-off, RHS: right heel strike, and RTO: right toe-off).

To identify the occurrence of heel strike and toe-off events, the relative distance between the hip and foot index was calculated in pixels, horizontally (Figure 7). In Figure 7, the heel strike events were indicated by the circle markers which represented the peak/maximum relative distance between the hip and foot index while the toe-off events were indicated by cross markers which represented the minima/minimum relative distance between the hip and foot index. To avoid the misidentification of heel strike and toe-off events, a time threshold was set at 0.8 s. At the same time, a peak was only detected when its value was larger or equal to 35% and 46% of the maximum relative distance between hip and foot index for the left and right legs, respectively, and the minimum was only detected when its value was smaller or equal to 18% of the minimum relative distance between hip and foot index for both legs. Based on the timing of the heel strike and toe-off events, the following temporal gait parameters were calculated and saved in a CSV file for each healthy individual:

(i) Stance time: the duration between heel strike and toe-off of the same leg.
(ii) Swing time: the duration between toe-off and heel-strike of the same leg.
(iii) Step time: the duration between consecutive heel strikes of both feet.
(iv) Double support time: the duration between the heel strike of one leg and the toe-off of the contralateral leg.

### 2.6. Statistics

Statistical analysis was conducted in IBM SPSS Statistics v26 to evaluate and validate the reliability and robustness of our system by comparing the temporal gait outcomes obtained from the Vicon motion capture system and our system. As the heel strike and toe-off events were the key gait events that aid the extraction of the temporal gait outcomes, descriptive statistics were conducted to compute the mean error, mean absolute error, and range of the mean error of heel strike and toe-off events timings obtained from the Vicon motion capture system and our system. Accordingly, descriptive statistics were conducted to compute the mean, standard deviation, mean error, and mean absolute error of the temporal gait parameters. Furthermore, to evaluate the statistically significant main effect, an independent samples t-test was performed. Correlation and absolute agreement between the two systems were then assessed using Pearson correlation coefficients (r) and intra-class correlation coefficients (ICC_(2,1)_), respectively. The level of significance for all analyses was set at 0.05. The performance of ICC_(2,1)_ was defined according to an accepted guideline that categorizes the result as poor (<0.500), moderate (0.500–0.750), good (0.750–0.900), and excellent (>0.900) [34]. Descriptive statistics, independent samples t-tests, Pearson correlation coefficients, and intra-class correlation coefficients were assessed for each gait cycle and the means of each healthy individual. Scatter plots of MediaPipe Pose versus the Vicon motion capture system were generated for the temporal gait parameters for each gait cycle and the means of each healthy individual.

## 3. Results

### 3.1. Descriptive Statistics for Key Gait Events (Heel Strike and Toe-Off)

In Table 1, the number of gait events detected by the Vicon moCap System is taken as the baseline for comparison. Our MediaPipe Pose-based system was able to identify 103 out of 106 heel strike events for the left leg (97.17%) and 101 out of 102 heel strike events for the right leg (99.02%). The MediaPipe Pose system also accurately detected 100 out of 102 toe-off events for the left leg (98.04%) and 100 out of 105 toe-off events for the right leg (95.24%). However, there were two instances where the system produced false detections—one for a left heel-strike event and one for a right toe-off event. In terms of differences between the Vicon moCap and MediaPipe systems, the mean error in identifying heel strike and toe-off events ranged from −4 ms to 20 ms, and the mean absolute error in these events ranged from 20 ms to 30 ms.

### 3.2. Statistical Analysis of Temporal Gait Parameters for All Gait Cycle 

The statistical analysis only considered complete gait cycles when assessing temporal gait parameters. Table 2 shows that the average error between the Vicon moCap and the MediaPipe Pose systems in temporal gait parameters, such as stance time, swing time, step time, and double support time (for each gait cycle), ranged from −20 ms to 40 ms. The mean absolute error in these parameters ranged from 30 ms to 50 ms.

**Figure 8:**
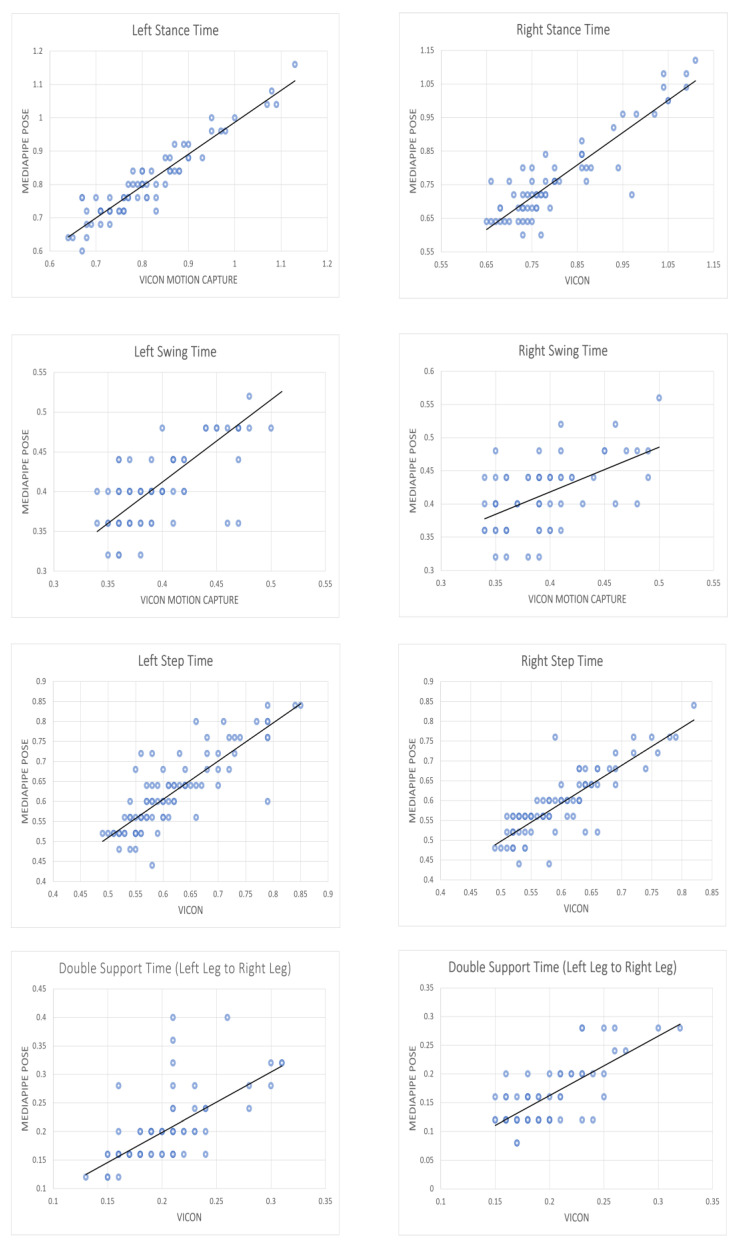
Scatter plots with linear fit of Vicon versus MediaPipe Pose walking temporal parameters for all gait cycles.

Table 3 indicates that no significant differences existed in stance time (left and right), swing time (left), step time (left and right), and double support time (left leg switched to right leg) between the two systems, as the significance (2-tailed) values from the independent samples t-tests exceeded the significance level of 0.05. However, significant differences existed in swing time (right) and double support time (right leg switched to left leg), as the significance (2-tailed) values were below 0.05. The Pearson correlation and intraclass correlation coefficient tests were significant at the 0.01 level (2-tailed) for all temporal gait parameters. For stance time (left and right) and step time (left and right), the Pearson correlation coefficient was good (ranging from 0.839 to 0.945), while it was moderate for swing time (left and right) and double support time (left leg switched to right leg and right leg switched to left leg) (ranging from 0.522 to 0.724). The intraclass correlation coefficient was excellent for stance time (left) (0.945), good for stance time (right) and step time (left and right) (ranging from 0.832 to 0.857), and moderate for swing time (left) and double support time (left leg switched to right leg and right leg switched to left leg) (ranging from 0.510 to 0.624) but weak for swing time (right) (0.469). The scatter plots in Figure 8 indicate that there was a linear increase in the temporal gait parameters obtained from the MediaPipe Pose system with those obtained from the Vicon motion capture system for all gait cycles. This suggested a positive association between the two systems, as shown in Figure 8. The correlation between the MediaPipe Pose system and the Vicon motion capture system was found to be strong for stance time and step time, but only moderate for swing time and double support time.

### 3.3. Statistical Analysis of Temporal Gait Parameters for the Means of Each Healthy Individual 

In this section, the statistical analysis assessed the means of temporal gait parameters for each healthy individual. Table 4 shows that the average error between the Vicon moCap and the MediaPipe Pose systems in temporal gait parameters, such as stance time, swing time, step time, and double support time (for each healthy individual means), ranged from −20 ms to 40 ms. The mean absolute error in these parameters ranged from 20 ms to 40 ms.

**Figure 9:**
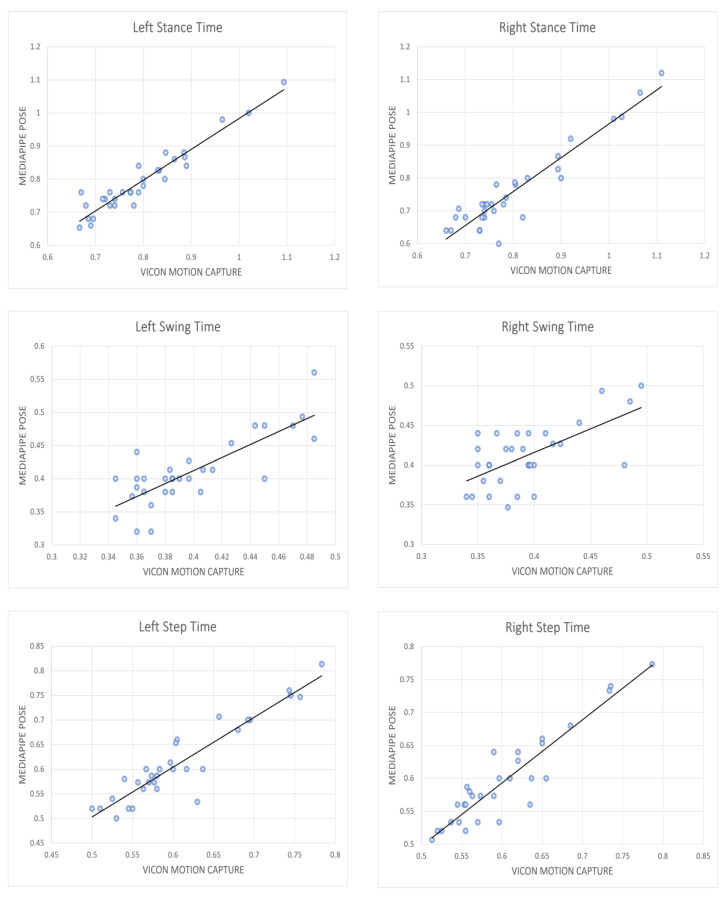

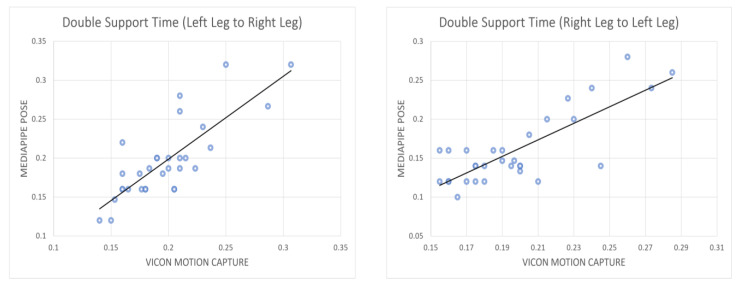
Scatter plots with linear fit of Vicon versus MediaPipe Pose walking temporal parameters for the means of each healthy individual.

Table 5 reveals that there were no significant differences in stance time (left and right), swing time (left and right), step time (left and right), and double support time (left leg switched to right leg) between the Vicon moCap and MediaPipe Pose systems, as the significance (2-tailed) values from the independent samples t-test were greater than the significance level of 0.05. However, there was a significant difference in double support time (right leg switched to left leg) between the Vicon moCap and MediaPipe Pose systems, as the significance (2-tailed) value was less than 0.05. The Pearson correlation and intraclass correlation coefficient tests were significant at the 0.01 level (2-tailed) for all temporal gait parameters. The Pearson correlation coefficient was good for all the temporal gait parameters (ranging from 0.802 to 0.955), except for swing time (right), which was rated as moderate (0.635). The intraclass correlation coefficient was excellent for stance time (left) and step time (left and right) (ranging from 0.923 to 0.956), good for stance time (right), swing time (left), and double support time (left leg switched to right leg) (ranging from 0.765 to 0.893), while it was moderate for swing time (right) and double support time (right leg switched to left leg) (ranging from 0.551 to 0.579). The scatter plots in Figure 9 indicate that there was a linear increase in the temporal gait parameters obtained from the MediaPipe Pose system with those obtained from the Vicon motion capture system for the means of each healthy individual. This suggested a positive association between the two systems, as shown in Figure 9. The correlation between the MediaPipe Pose system and the Vicon motion capture system was found to be strong for all temporal gait parameters, except left swing time, right swing time, and double support time (right leg switched to left leg).

## 4. Discussion

The aim of this study was to evaluate the accuracy and reliability of using the MediaPipe Pose model to provide an automated gait analysis without human intervention. The accuracy of this approach was compared to a three-dimensional Vicon motion capture system, which used ten motion capture cameras and built-in gait analysis software. This study had shown the potential of the application of markerless, automated gait analysis based on MediaPipe Pose to enable assessment for a wider range of individuals.

### 4.1. Performance of MediaPipe Pose

The BlazePose GHUM Heavy model of MediaPipe Pose was utilized to obtain precise body key points locations in this research. Nevertheless, this led to an increase in inference latency, which resulted in MediaPipe Pose estimating the body key points’ location with an average inference speed of 9 fps on the CPU. The reason MediaPipe Pose was chosen over other pose estimation models such as OpenPose and PoseNet is that it employs a top-down approach, where human candidates are first detected by a human detector, and then single-person pose estimation is performed. This approach yields more accurate key point detection than a bottom-up approach where key points are predicted all at once and then assembled into full poses for all individuals. Although the top-down approach is time-consuming because the pose of each person is estimated independently and the inference time is proportional to the number of detected persons, MediaPipe Pose is a single-person pose estimator model and human detection is not performed in each frame, thus enabling faster inference [31].

### 4.2. Temporal Gait Parameters Assessment

The study found that the MediaPipe Pose system has the potential for quantitative temporal analysis of gait and is suitable for clinical and biomechanical assessments of human walking. Based on the descriptive statistics, the system has shown low mean absolute error in assessing temporal gait parameters for all gait cycles and the means of each healthy individual.

**Figure 10:**
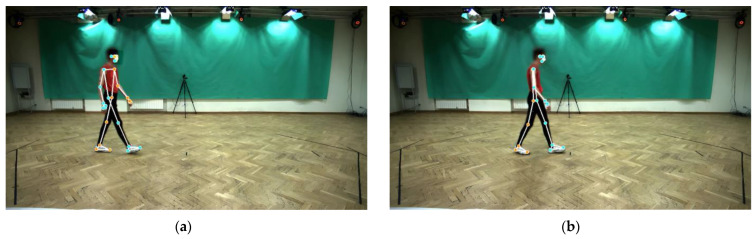
(**a**) Correct identification of left and right lower limbs (orange markers: left lower limb, blue markers: right lower limb) (**b**) Misidentification of left and right lower limbs (orange markers: right lower limb, blue markers: left lower limb).

Overall, the statistical test result for the means of each healthy individual is better compared to the statistical test result for all gait cycles. For each healthy individual means, the independent samples t-test results have shown that there is no significant difference in the temporal gait parameters between the MediaPipe Pose and Vicon moCap system except for double support time (right leg switched to left leg). The Pearson correlation coefficient is satisfactory for all temporal gait parameters, except for swing time (right) which was rated as moderate. The intraclass correlation coefficient was good for stance time (left and right), step time (left and right), swing time (left), and double support time (left leg switched to right leg), while moderate for swing time (right) and double support time (right leg switched to left leg). This is because the duration of the double support times is very short (0.20 s). Thus, it is hard for the Mediapipe Pose system to calculate the double support time using the input video recorded at low frames per second, 25 fps. In addition, the misidentification of the left and right lower limbs by the MediaPipe Pose system (Figure 10) affects the accuracy of the temporal gait parameters assessment. This effect is minimized through data filtering techniques but still has an impact on the accuracy of gait events (heel strike and toe-off) identification, which is important for further extraction of temporal gait parameters. 

### 4.3. Qualitative Comparison with Other Works

Table 6 presents gait analysis performed using Azure Kinect and Kinect v2, which can evaluate spatiotemporal gait parameters such as step time, step length, step width, stride length, and stride time. The relative error for spatial gait parameters ranges from −0.001 m to 0.040 m, while the relative error for temporal gait parameters ranges from 0.000 s to 0.010 s. In our study, we propose a gait analysis method based on a markerless pose estimation model (MediaPipe Pose) that can assess spatiotemporal gait parameters including stance time, swing time, step time, and double support time. The relative error for temporal gait parameters in our analysis ranges from −0.02 s to 0.02 s. Both the gait analysis using Azure Kinect and Kinect v2, as well as our proposed method, demonstrate low relative errors in assessing spatiotemporal gait parameters.

Nevertheless, our approach attains similar spatiotemporal gait parameters even at a lower video resolution and frame rate when compared to the gait analysis conducted with Azure Kinect and Kinect v2. It is important to acknowledge that the limited video frame rate of 30 fps in Azure Kinect and Kinect cameras imposes restrictions on their suitability for gait assessment during faster walking or running scenarios. Conversely, our method allows for gait assessment in faster walking or running conditions by leveraging videos recorded at higher frame rates using high-speed cameras.

In the future, our study will broaden its scope to include cerebellar ataxia patients. The observed gait differences in cerebellar ataxia patients during preferred paced walking, when compared to healthy individuals, include reduced walking speed, cadence, step length, stride length, and swing phase, as well as increased base width, stride time, step time, stance phase, and double limb support phase. Additionally, there will be an evident increase in variability within step and stride parameters. Among these parameters, the most significantly affected ones in cerebellar ataxia are speed, double limb support phase duration, and variability in stride time [35]. Thus, our approach focuses on evaluating novel spatiotemporal gait parameters, such as stance time, swing time, step time, and double support time, in healthy individuals as a basis for future comparison with cerebellar ataxia patients. 

**Table 6:** Spatiotemporal gait parameter comparison with other works.

Method	Video Resolution	Video Frame Rate	Spatiotemporal Gait Parameter (Relative Error)								Year	Ref.
			Stance Time (s)	Swing Time (s)	Step Time (s)	Double Support Time (s)	Step Length (m)	Step Width (m)	Stride Length (m)	Stride Time (s)		
Azure Kinect	3840 × 2160 px	30 fps	✕	✕	✕	✕	−0.03	−0.001	−0.04	0.01	2022	[36]
Azure Kinect	3840 × 2160 px	30 fps	✕	✕	0.000	✕	0.00	0.040	✕	0.00	2020	[37]
Kinect v2	1920 × 1080 px	30 fps	✕	✕	0.000	✕	−0.05	−0.070	✕	0.00	2020	[37]
Our Work	960 × 540 px	25 fps	0.02	−0.02	0.001	0.02	✕	✕	✕	✕		

Note: ✕ denotes the data is not available in the cited reference.

### 4.4. Implications of the Proposed Approach in Clinical Settings

Automated gait analysis based on MediaPipe Pose has the potential to improve the diagnosis and treatment of gait abnormalities in clinical settings. The analysis can help identify gait abnormalities at an early stage, providing an objective way to assess gait and track progress, and tailoring treatment interventions to the specific needs of each patient. Automated gait analysis can also be a cost-effective alternative to traditional gait analysis methods, making it more accessible to a wider range of patients. Remote monitoring of gait using automated analysis can be used to improve patient compliance with treatment and reduce the burden on healthcare providers. 

However, it is essential to verify the effectiveness of automated gait analysis based on pose estimation in various clinical populations, including both adults and children. Previous research has indicated that current pose estimation algorithms can accurately track the gait of patient populations using walking aids [38], but tracking patient populations who use prosthetic devices that differ from those used to train the algorithms presents challenges [39]. Therefore, it is critical to validate the accuracy and reliability of automated gait analysis in diverse patient populations before implementing it in clinical settings. Although this study used a pre-trained network [18], utilizing a network specifically trained for gait and clinical conditions may improve the accuracy of the results.

## 5. Conclusions

The automated temporal gait analysis based on a markerless pose estimation model (MediaPipe Pose) can be used to calculate temporal gait parameters, including stance time, swing time, double support time, and step time, with low mean absolute error without any human intervention. The approach exhibits excellent intraclass correlation coefficients for stance time (left) and step time (left and right) (0.923 to 0.956), good intraclass correlation coefficients for stance time (right), swing time (left), and double support time (left leg switched to right leg) (0.765 to 0.893), and moderate intraclass correlation coefficients for double support time (right leg switched to left leg) and swing time (right) (0.551 to 0.579). These parameters are essential for monitoring changes in gait and assessing the efficacy of interventions such as rehabilitation or training programs. The method is cost-effective and accessible compared to instrumented gait analysis, making it possible to conduct large-scale gait analysis in different populations. Additionally, it enables tracking of gait patterns in real-life situations, providing more naturalistic validity and a better understanding of gait irregularities during daily activities.

## 6. Limitations and Future Work

At present, a markerless MediaPipe Pose model-based automated gait analysis system has achieved a satisfactory level of accuracy in detecting left and right heel strike and toe-off events during gait analysis, with detection rates ranging from 95.24% to 99.02%. However, the system produces two false detections, resulting in some missing and inaccurate temporal gait parameter calculations. To improve the system, future work will explore better alternative approaches such as moving averages to identify peaks (heel strike event) and minima (toe-off event). Additionally, the walking sequence of healthy individuals could be captured at a higher resolution and frame rate to minimize the misidentification of lower limbs caused by fast walking speeds, resulting in more precise identification of gait events.

Moreover, the proposed approach could be further enhanced by incorporating spatial gait parameters and lower limb joint kinematics, making it a powerful tool for pathology evaluation. For instance, by examining the variability of spatial gait parameters, temporal gait parameters, and spatiotemporal gait parameters, it is possible to evaluate the progression of Friedreich ataxia [40]. Additionally, the spatiotemporal parameters and lower extremity kinematics during the gait cycle of adult patients with cervical spondylotic myelopathy differ from those of healthy individuals. By identifying the relationship between abnormal spinal alignment and lower extremity function, as well as the specific gait and biomechanical issues that myelopathic patients experience, clinicians can gain a better understanding of the disease and develop more effective rehabilitation protocols [41].

## Figures and Tables

**Table 1:** Heel strike and toe-off events timings identified for each gait cycle.

Gait Event	Leg	N	TP	FP	Mean ± SD (V—MP)	Mean ± SD (|V—MP|)	Range (V—MP)
Heel strike time	L	106	103	1	−0.004 ± 0.03	0.02 ± 0.02	[−0.13, 0.09]
	R	102	101	0	0.02 ± 0.05	0.03 ± 0.04	[−0.17, 0.20]
Toe-off time	L	102	100	0	−0.004 ± 0.03	0.02 ± 0.03	[−0.10, 0.18]
	R	105	100	1	0.005 ± 0.04	0.02 ± 0.03	[−0.11, 0.17]

Note: V represents Vicon moCap and MP represents MediaPipe Pose, N represents the number of gait events detected by Vicon moCap, TP represents the number of true gait events detected by MediaPipe Pose, and FP represents the number of false gait events detected by MediaPipe Pose.

**Table 2:** Descriptive statistics of temporal gait parameters calculated for each gait cycle.

Temporal Gait Parameter	Leg	N	Mean ± SD		Mean ± SD (V—MP)	Mean ± SD (|V—MP|)
			V	MP		
Stance time	L	71	0.81 ± 0.11	0.80 ± 0.11	0.01 ± 0.04	0.03 ± 0.02
	R	65	0.81 ± 0.12	0.77 ± 0.13	0.04 ± 0.06	0.05 ± 0.04
Swing time	L	69	0.40 ± 0.04	0.41 ± 0.06	−0.01 ± 0.05	0.03 ± 0.04
	R	65	0.39 ± 0.04	0.41 ± 0.05	−0.02 ± 0.05	0.04 ± 0.03
Step time	L	88	0.62 ± 0.09	0.62 ± 0.10	−0.005 ± 0.05	0.04 ± 0.04
	R	82	0.60 ± 0.07	0.59 ± 0.08	0.01 ± 0.04	0.03 ± 0.03
Double support time	L2R	71	0.20 ± 0.04	0.20 ± 0.07	0.001 ± 0.05	0.03 ± 0.04
	R2L	62	0.20 ± 0.04	0.16 ± 0.05	0.04 ± 0.04	0.04 ± 0.03

Note: V represents Vicon moCap, MP represents MediaPipe Pose, N represents number of gait parameters assessed, L2R represents left leg switched to right leg, and R2L represents right leg switched to left leg.

**Table 3:** Statistical tests for temporal gait parameters calculated for each gait cycle.

Temporal Gait Parameter	Leg	Significance (2-Tailed)	Pearson Correlation, r	ICC_(2,1)_
Stance time	L	0.785	0.945	0.945
	R	0.075	0.900	0.857
Swing time	L	0.199	0.690	0.624
	R	0.020	0.522	0.469
Step time	L	0.737	0.839	0.832
	R	0.579	0.853	0.846
Double support time	L2R	0.891	0.625	0.552
	R2L	<0.001	0.724	0.510

Note: V represents Vicon moCap, MP represents MediaPipe Pose, r represents Pearson correlation, ICC_(2,1)_ represents intraclass correlation coefficient for absolute agreement, L2R represents left leg switched to right leg, and R2L represents right leg switched to left leg.

**Table 4:** Descriptive statistics of temporal gait parameters calculated for the means of each healthy individual.

Temporal Gait Parameter	Leg	N	Mean ± SD		Mean ± SD (V—MP)	Mean ± SD (|V—MP|)
			V	MP		
Stance time	L	31	0.80 ± 0.10	0.79 ± 0.10	0.003 ± 0.03	0.02 ± 0.02
	R	31	0.81 ± 0.12	0.76 ± 0.13	0.04 ± 0.04	0.04 ± 0.04
Swing time	L	31	0.40 ± 0.04	0.41 ± 0.05	−0.01 ± 0.03	0.03 ± 0.02
	R	31	0.39 ± 0.04	0.41 ± 0.04	−0.02 ± 0.03	0.03 ± 0.02
Step time	L	31	0.61 ± 0.08	0.61 ± 0.08	−0.005 ± 0.03	0.02 ± 0.02
	R	31	0.60 ± 0.07	0.59 ± 0.07	0.01 ± 0.03	0.02 ± 0.02
Double support time	L2R	31	0.20 ± 0.04	0.20 ± 0.05	0.001 ± 0.03	0.02 ± 0.02
	R2L	31	0.20 ± 0.03	0.16 ± 0.05	0.04 ± 0.03	0.04 ± 0.03

Note: V represents Vicon moCap, MP represents MediaPipe Pose, N represents the number of gait parameters assessed, L2R represents left leg switched to right leg, and R2L represents right leg switched to left leg.

**Table 5:** Statistical tests for temporal gait parameters calculated for the means of each healthy individual.

Temporal Gait Parameter	Leg	Significance (2-Tailed)	Pearson Correlation, r	ICC_(2,1)_
Stance time	L	0.922	0.955	0.956
	R	0.196	0.944	0.893
Swing time	L	0.306	0.802	0.765
	R	0.076	0.635	0.579
Step time	L	0.824	0.931	0.928
	R	0.692	0.926	0.923
Double support time	L2R	0.902	0.804	0.779
	R2L	0.001	0.805	0.551

Note: V represents Vicon moCap, MP represents MediaPipe Pose, r represents Pearson correlation, ICC_(2,1)_ represents intraclass correlation coefficient for absolute agreement, L2R represents left leg switched to right leg, and R2L represents right leg switched to left leg.

## Data Availability

The GPJATK dataset that featured synchronized and calibrated video from multiple angles and motion capture is accessible to the public at http://bytom.pja.edu.pl/projekty/hm-gpjatk/ (accessed on 9 September 2022) [32]. The GPJATK Dataset Release Agreement has been signed to use this dataset in this study.
